# Abundant supernova dust and heterogeneous aqueous alteration revealed by stardust in two lithologies of asteroid Bennu

**DOI:** 10.1038/s41550-025-02688-3

**Published:** 2025-12-02

**Authors:** Ann N. Nguyen, Laura B. Seifert, Kei Shimizu, Kathie Thomas-Keprta, Loan Le, Lindsay P. Keller, Simon J. Clemett, Zia Rahman, Jessica J. Barnes, Harold C. Connolly, Dante S. Lauretta

**Affiliations:** 1 Astromaterials Research and Exploration Science Division, NASA Johnson Space Center, Houston, TX USA; 2 Amentum/JETS II, NASA Johnson Space Center, Houston, TX USA; 3 Barrios/JETS II, NASA Johnson Space Center, Houston, TX USA; 4 Asterion/JETS II, NASA Johnson Space Center, Houston, TX USA; 5 Lunar and Planetary Laboratory, University of Arizona, Tucson, AZ USA; 6 Department of Geology, Rowan University, Glassboro, NJ USA; 7 Department of Earth and Planetary Sciences, American Museum of Natural History, New York, NY USA

**Keywords:** Asteroids, comets and Kuiper belt, Astrophysical dust, Laboratory astrophysics, Early solar system, Mineralogy

## Abstract

The oldest constituents in chondritic samples are presolar grains that condensed in the outflows and explosions of dying stars. These grains divulge the types and concentrations of dust that seeded our Solar System. However, they are subject to destruction during planetesimal formation and alteration. We conducted a detailed study of presolar grains in fragments of asteroid Bennu to elucidate the alteration history of distinct lithologies—angular and hummocky—and the sources of stardust that were accreted by the parent asteroid. The presolar grain abundances support a history of substantial aqueous alteration. Nevertheless, we found organic-rich clasts within a hummocky particle having higher presolar silicate abundances, akin to some of the least altered chondritic meteorites, and presolar silicate, oxide and SiC grains that retain their crystallinity. These clasts illustrate that aqueous alteration was heterogeneous within the parent body and their properties may better represent the starting materials that accreted to form the protolith. In addition, the Bennu samples we analysed have a six-times greater proportion of C-rich supernova dust than other chondritic samples, injected perhaps from a nearby supernova. This observation adds to evidence that Bennu’s parent body sampled a region of the protoplanetary disk having a distinct mixture of starting materials.

## Abstract (web summary)

Bennu samples have abundant supernova stardust and clasts that are richer in presolar silicates and organics than other chondritic samples, suggesting that the protolith sampled material with a unique mixture of primordial components before undergoing heterogeneous aqueous alteration.

## Main

Presolar stardust grains are found at trace levels (parts per million) in meteorites, interplanetary dust particles (IDPs), Antarctic micrometeorites (AMMs), comet 81 P/Wild2 samples returned by NASA’s Stardust mission, and carbonaceous asteroid (162173) Ryugu samples returned by JAXA’s Hayabusa2 mission^1–4^. Their highly anomalous isotopic compositions result from nucleosynthetic reactions in evolved red giant stars, supernovae and novae. The mineralogy and chemistry of presolar grains can be used to constrain condensation conditions and to probe the effects of secondary alteration, as these grains are susceptible to alteration or destruction in space, in the solar nebula and within planetesimals. Specifically, presolar silicates are highly sensitive to hydration, whereas presolar SiC grains are altered through heating and oxidation^1,5^. The investigation of presolar grains can therefore provide constraints on the provenance and geologic history of their asteroidal and cometary hosts.

NASA’s Origins, Spectral Interpretation, Resource Identification and Security–Regolith Explorer (OSIRIS-REx) mission returned 121.6 g of pristine regolith from the carbonaceous asteroid (101955) Bennu^6^. Bennu is a rubble pile asteroid, consisting of fragments of a larger parent body that was collisionally disrupted (see, for example, ref. ^7^). Spacecraft observations have shown that Bennu’s surface material is hydrated and has carbonate veins, which indicate that large-scale fluid flow occurred within the parent asteroid^8,9^. Indeed, the returned samples are dominated by secondary phases that include phyllosilicate minerals, magnetite, carbonate, sulfides and phosphates^6,10–12^. However, primary constituents have also been identified, including anhydrous minerals from the early Solar System, and organics and presolar grains from beyond the Solar System^6,13^.

These presolar grains were identified among loose, unsorted (aggregate) material. However, petrographic analyses of the returned samples distinguished at least two lithologies—dubbed angular and hummocky—that may correspond to distinct boulder types observed on Bennu^6,10^. Here we perform a detailed study of presolar grains in angular and hummocky particles of Bennu to elucidate the stellar sources of presolar materials accreted by the parent asteroid and to understand the alteration histories of the different lithologies. We compare the presolar grain inventory in Bennu with those of chondritic samples, in particular, Ivuna-type (CI) and Mighei-type (CM) carbonaceous chondrites, and Ryugu, to further explore potential linkages and to constrain the reservoir from which Bennu’s protolith accreted.

## Results

### Chemical characterization

**Extended Data Fig. 1:**
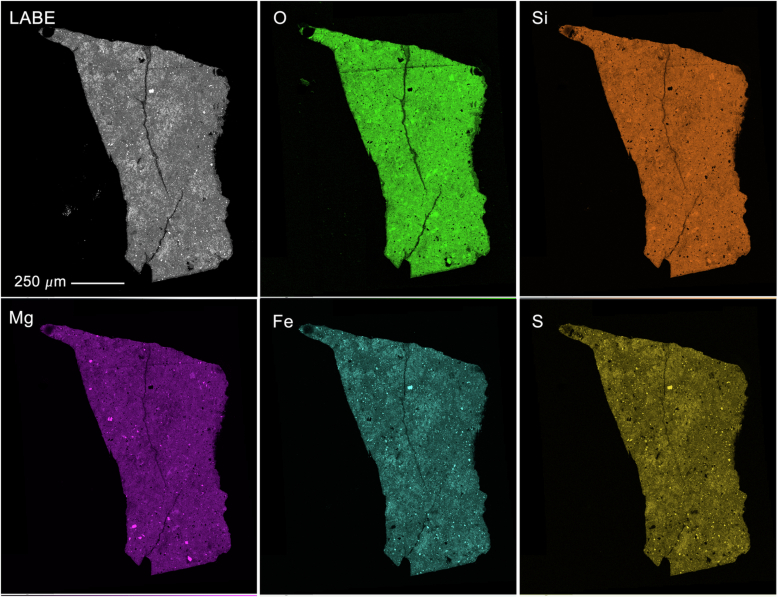
Low-angle backscattered electron (LABE) image of angular particle OREX-803165-0 and corresponding elemental maps. The chemical composition shows some variation across the particle, but well-defined clasts are not present.

**Extended Data Fig. 2:**
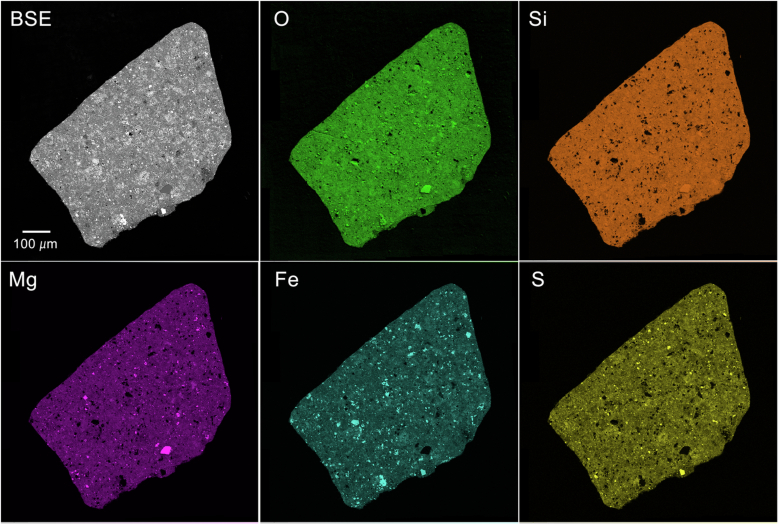
BSE image and elemental maps of angular particle OREX-803170-0. Similar to angular particle OREX-803165-0, this particle does not contain well-defined clasts and is more chemically homogeneous.

Polished sections of ~millimetre-sized Bennu particles were characterized for their chemistry and petrography by field emission–scanning electron microscopy and energy-dispersive X-ray spectroscopy (FE-SEM-EDX; Methods). We analysed two angular particles (OREX-803165-0 and OREX-803170-0), which are dominated by a fine-grained phyllosilicate matrix with numerous magnetite framboids and pyrrhotite crystals, and minor apatite and magnesite (see also ref. ^10^). Rare phases include dolomite, calcite, pentlandite, ilmenite, chromite and phosphides. OREX-803165-0 contains one region of coarse-grained phyllosilicates. Several anhydrous Mg-rich olivine grains are present in both angular particles, and two Mg,Al-spinel grains were observed in OREX-803170-0. The elemental maps show that the matrix is relatively homogeneous, with a few regions having slight enrichments in Fe and S and depletions in Si and Mg (Extended Data Figs. 1 and 2).

**Fig. 1:**
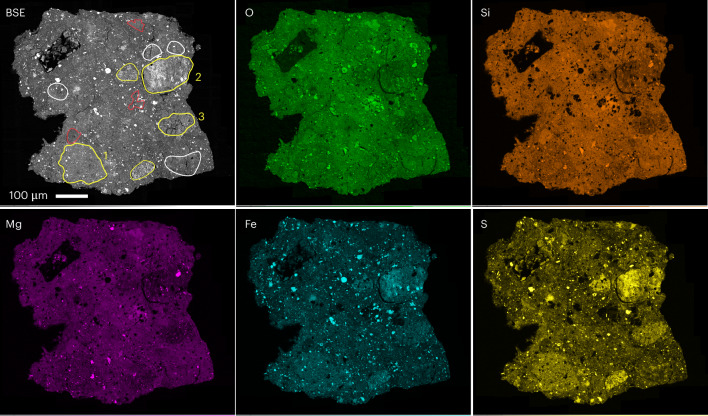
BSE image of a fragment of hummocky particle OREX-803172-0 and corresponding elemental maps. The different types of clasts are outlined in yellow (type (i): S rich), red (type (ii): Si rich) and white (type (iii): nodules chemically similar to matrix). The yellow-outlined clasts labelled 1–3 are enriched in Fe and S and depleted in Mg and Si.

**Extended Data Fig. 3:**
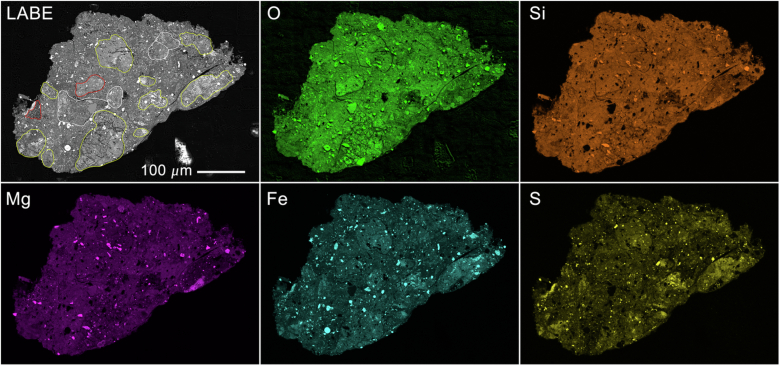
LABE image of a fragment of hummocky particle OREX-803172-0 and corresponding elemental maps. Outlined in yellow are S-rich clasts (type i), red are Si-enriched clasts (type ii), and white are chemically similar to the matrix but appear to be physically distinct (type iii).

We analysed one hummocky particle (OREX-803172-0); it shares similar components with the angular particles but contains more spinel, dolomite and calcite and less apatite. Pyrrhotite having a fibrous texture and enstatite are also observed. Hummocky Bennu particles are breccias that are distinguished by their rough morphology and clastic nature, exemplified by rounded clasts having well-defined boundaries with the surrounding matrix (Fig. 1 and Extended Data Fig. 3). The clasts in particle OREX-803172-0 are ~30–175 μm in size. We observe three types based on backscattered electron (BSE) images and intensity variations in the SEM-EDX maps: type (i), the most abundant type, appears brighter in BSE images and is rich in S and Fe and poor in Si and Mg relative to the bulk groundmass and other clast types; type (ii) is smaller, Si rich and dominated by coarse-grained phyllosilicates; and type (iii) is chemically indistinguishable from the bulk matrix but occurs as separate rounded nodules.

### Nanometre-scale isotope mapping

In situ isotope mapping was conducted using nanoscale secondary ion mass spectrometry (NanoSIMS; Methods) on matrix regions and clasts in the Bennu particles. Clasts measured in hummocky particle OREX-803172-0 included eight of type (i) (S rich), one of type (ii) (Si rich) and three of type (iii) (matrix like).

**Fig. 2:**
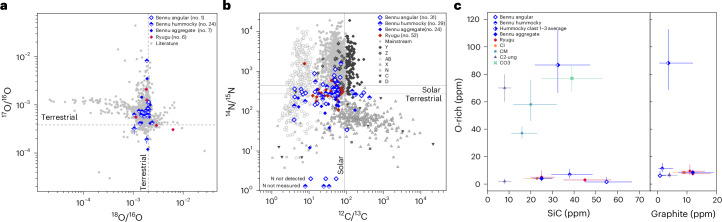
Isotopic compositions and abundances of presolar grains in angular particles OREX-803165-0 and OREX-803170-0 and hummocky particle OREX-803172-0. **a**, O isotopic compositions of presolar silicates and oxides. **b**, C and N isotopic compositions of presolar SiC grains. Presolar grains from Bennu aggregate samples^6,13^, Ryugu samples^1,2^ and the presolar grain database^14,15^ are shown for comparison. In the legends, the number of grains identified in Bennu and Ryugu samples are given in parentheses. For clarity, error bars (1*σ*) are only shown in **a** for data acquired in this study. All errors can be found in Supplementary Tables 1 and 2. **c**, Abundances of presolar phases in the matrix of the Bennu particles and the mean abundance in three less-altered clasts from within the hummocky particle. Shown for comparison are data from Bennu aggregate^13^ and Ryugu samples^1,2^, and from the chondrites Tagish Lake and Adelaide (C2-ung), Ivuna (CI), Asuka 12236 (CM2.9), Paris (CM2.7–2.9) and MIL 090019 (CO3)^2,28–30,33,53^. Errors are 1*σ*.

Among the three particles, we found a total of 63 C-rich grains and 25 O-rich grains having highly anomalous C, N, O or Si isotopic compositions. These isotopic compositions fall within the ranges reported in the literature for presolar grains^4,14,15^ (Fig. 2a, b and Supplementary Tables 1 and 2).

**Extended Data Fig. 4:**
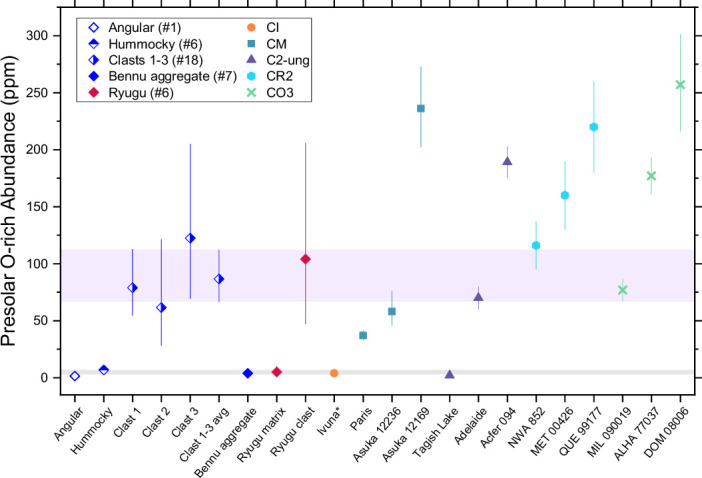
Abundances of O-rich presolar grains. Abundances in the different Bennu samples analyzed here are compared with those in an aggregate Bennu sample, matrix material and an exogenous clast from Ryugu, and various carbonaceous chondrites. The abundance for Ivuna is an upper limit. See Methods for supporting references. The grey shaded region is the mean abundance for Bennu angular, aggregate, and hummocky samples, excluding Clasts 1–3 (see Source Data). The purple shaded region is the mean abundance for Clasts 1–3 from the hummocky Bennu particle, including 1σ errors. Error bars are 1σ.

**Extended Data Fig. 5:**
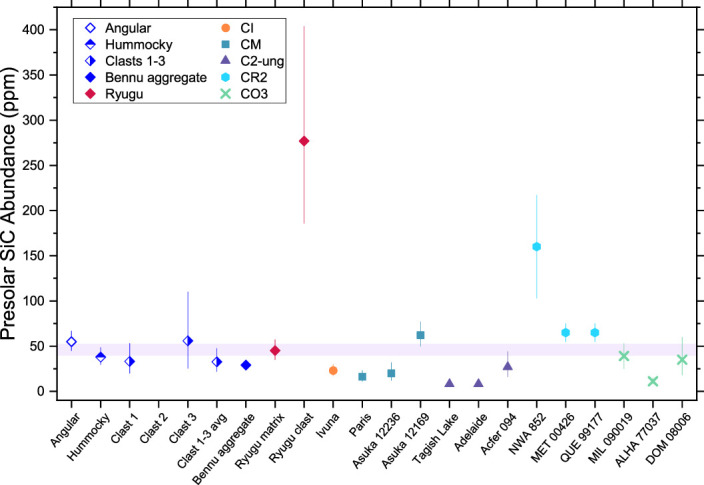
Abundances of presolar SiC grains. As in Extended Data Fig. 4 but for presolar SiC grains. No presolar SiC grains were identified in Clast 2. See Methods for supporting references. The mean abundance for Bennu, including the clasts, is shown as the purple shaded region, including 1σ errors (see Source Data). Error bars are 1σ.

In the angular particles, only one presolar oxide (325 nm in diameter) and no presolar silicate grains were identified. We observed 32 C-rich presolar grains, of which 31 are SiC and 1 is graphite. The average size of the C-rich grains is 330 nm. The abundance of O-rich presolar grains in the two angular particles is ${2}_{-1}^{+4}$ ppm (1*σ*) (Fig. 2c, Extended Data Fig. 4 and Extended Data Table 1). The abundances of presolar SiC and presolar graphite are ${55}_{-10}^{+12}$ and ${1}_{-1}^{+2}$ ppm, respectively (Fig. 2c, Extended Data Fig. 5 and Extended Data Table 1).

In the hummocky particle, 21 presolar silicates and 3 presolar oxides were identified, with an average size of 295 nm. In addition, 29 presolar SiC and 2 presolar graphite grains were detected, having an average size of 305 nm. The abundance of O-rich presolar grains is heterogeneous across the hummocky particle, with most (75%) located within three S-rich clasts (labelled in Fig. 1). These clasts have higher abundances (79–122 ppm; ${87}_{-20}^{+26}$ ppm total) than the bulk of the hummocky particle (${7}_{-3}^{+4}$ ppm) (Fig. 2c, Extended Data Fig. 4 and Extended Data Table 1). The abundance of C-rich presolar grains is consistent across the particle, with total abundances of ${36}_{-7}^{+8}$ ppm for SiC and ${2}_{-1}^{+3}$ ppm for graphite (Fig. 2c, Extended Data Fig. 5 and Extended Data Table 1).

### Microstructure of presolar grains

The chemical compositions and crystal structures of six presolar grains and the surrounding material were determined by scanning and transmission electron microscopy (STEM and TEM; Methods), EDX mapping and selected-area electron diffraction (SAED). We analysed three grains from ‘Clast 1’, one grain from ‘Clast 2’ and two grains from ‘Clast 3’ in hummocky particle OREX-803172-0 (Fig. 1). These clasts are type (i) and have elevated abundances of O-rich presolar grains (Extended Data Fig. 4).

**Fig. 3:**
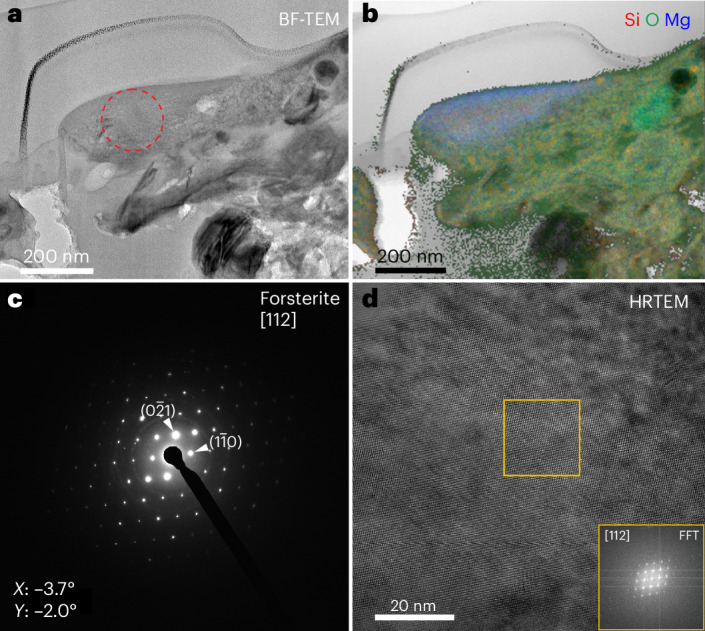
Characterization of a crystalline presolar forsterite grain (16_2) preserved in Clast 1 of hummocky particle OREX-803172-0. **a**, Bright-field TEM image of the presolar grain and surrounding matrix. **b**, False-colour RGB composite EDX map of Si, O and Mg. **c**, SAED pattern obtained from the region denoted by the dashed red circle in **a** at tilt angles *X* = –3.7°, *Y* = –2.0°. **d**, High-resolution TEM image of 16_2 and inset of the fast Fourier transform (FFT) from the region denoted by the orange box. The SAED pattern and FFT are consistent with the [112] zone axis of forsterite.

The ^17^O-rich grain 16_2 from Clast 1 measures ~600 × 160 nm (Fig. 3 and Supplementary Table 1). It is a single crystal of forsterite (Fo_99_; Mg_2_SiO_4_). The matrix within the same focused ion beam (FIB) cross-section consists of a fine-grained phyllosilicate groundmass with Fe and Fe–Ni sulfides, organic nanoglobules, a Ca pyroxene grain and an Fe, Zn sulfide.

**Fig. 4:**
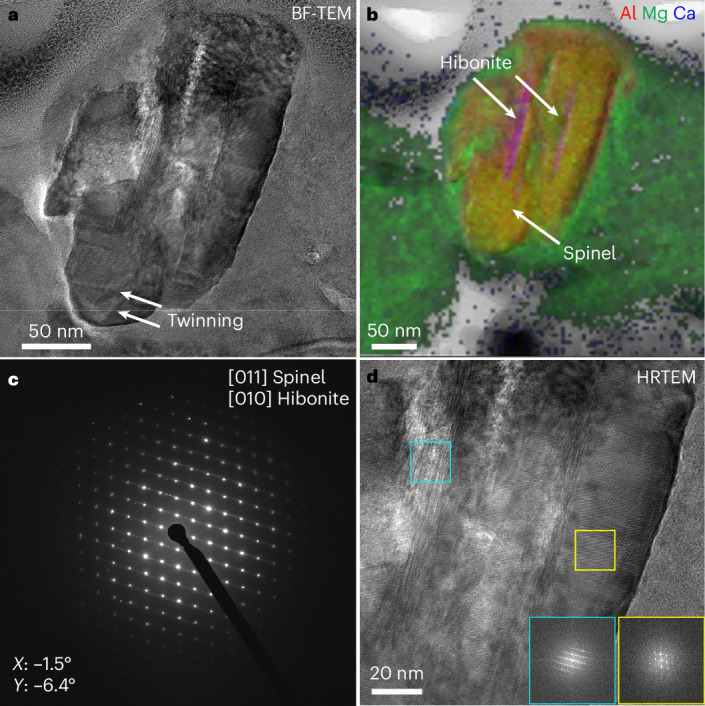
Characterization of a presolar spinel–hibonite grain (19_1) from Clast 1 of hummocky particle OREX-803172-0. **a**, Bright-field TEM image of 19_1 with twinning indicated by the white arrows. **b**, False-colour RGB composite EDX map of Al, Mg and Ca. Regions containing spinel and hibonite are labelled. **c**, SAED pattern obtained at tilt angles *X* = −1.5°, *Y* = −6.4°, showing the crystallographic orientation relationship of the [011] zone axis of spinel and the [010] zone axis of hibonite. **d**, High-resolution TEM image of the spinel–hibonite assemblage with associated FFT insets for [011] spinel (yellow box) and [010] hibonite (cyan box).

The ^17^O-rich grain 19_1 (Fig. 4) from Clast 1 measures ~250 × 145 nm. It is a spinel grain with minor Cr (MgAl_2_O_4_,0.8 wt% Cr) and two central Ca- and Al-bearing hibonite laths. The diffraction data demonstrate a crystallographic orientation relationship between the spinel and hibonite: [010]_h__ibonite_ // [011]_s__pinel_. Twinning is also observed in one portion of the spinel. The surrounding matrix is dominated by fine-grained phyllosilicates with Fe and Fe–Ni sulfides, one carbonate and one anhydrous silicate.

**Extended Data Fig. 6:**
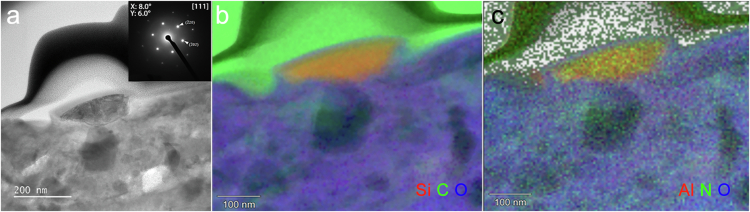
TEM analysis of a presolar SiC grain (15c) in Clast 1 of particle OREX-803172-0. (**a**) BF-TEM image of the presolar grain under C and Pt caps which served to mark the grain. The inset is a SAED pattern obtained at tilt angles X = 8.0°, Y = 6.0°, demonstrating that the SiC grain is the 3C polytype. (**b**) False-color composite elemental map of Si, C, and O showing the SiC grain has an oxidized rim. (**c**) False-color composite elemental map of Al, N, and O, showing that grain 15c contains Al and N.

A ^15^N-poor, ^13^C-rich presolar grain (15c) from Clast 1 is an ~315 × 60 nm-sized single SiC crystal of the 3C polytype (Extended Data Fig. 6 and Supplementary Table 2). The grain contains Al and N and is surrounded by an oxidized rim.

**Extended Data Fig. 7:**
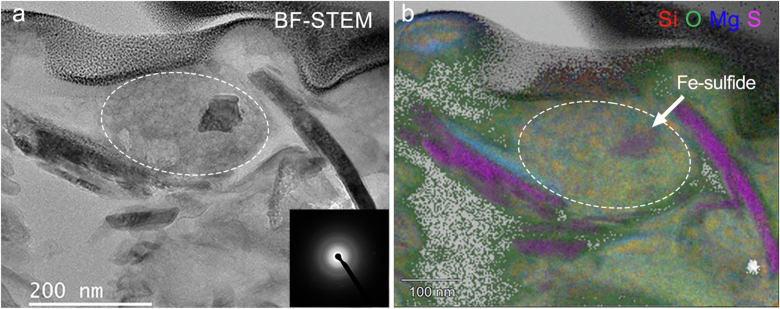
TEM analysis of a presolar silicate grain (5_3b) in Clast 2 of particle OREX-803172-0. (**a**) BF-STEM image of the presolar silicate grain (circled). The inset is a SAED pattern demonstrating that the silicate grain is amorphous. **b**) Composite elemental map of the presolar silicate grain showing an Fe-sulfide inclusion.

The ^17^O-rich, ^18^O-poor grain 5_3b from Clast 2 measures ~270 × 150 nm and is an amorphous, non-stoichiometric grain with composition similar to pyroxene (Extended Data Fig. 7). The grain contains a 50-nm crystalline Fe sulfide inclusion with a composition consistent with pyrrhotite. The matrix immediately surrounding 5_3b contains a lath-shaped spinel grain and Fe sulfides. The remaining matrix in the FIB section is dominated by fine-grained phyllosilicates, Fe and Fe–Ni sulfides and nanoglobules.

**Extended Data Fig. 8:**
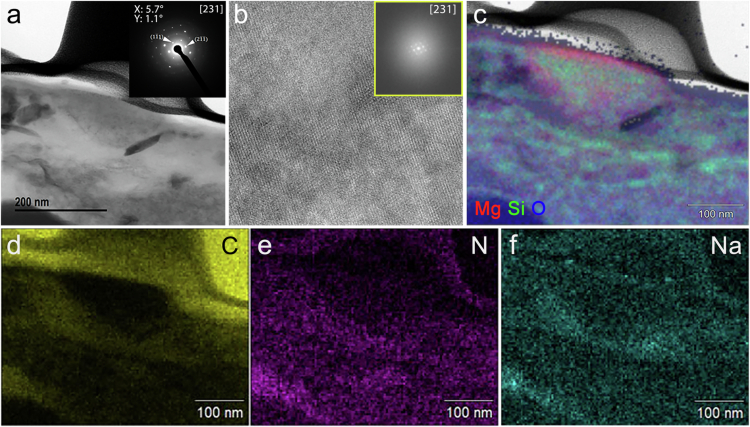
TEM analysis of a presolar silicate grain (2b_2a) in Clast 3 of particle OREX-803172-0. (**a**) BF-TEM image of the presolar grain under C and Pt caps which served to mark the grain. The inset is a [231] zone-axis SAED pattern obtained at tilt angles X = 5.7°, Y = 1.1°, consistent with orthopyroxene. (**b**) HRTEM image and inset of the FFT for [231] orthopyroxene. (**c**) Composite Mg, Si, and O elemental map of the pyroxene grain showing a partial Mg-rich rim. (**d–f**) Elemental maps showing C-, N-, and Na-bearing material in the section, including directly surrounding the presolar grain.

Grain 2b_2a from Clast 3 is ^17^O-rich and ^18^O-poor and measures ~250 × 105 nm. Its composition is consistent with pyroxene (MgSiO_3_) with a Mg enrichment at the top and left edges (Extended Data Fig. 8). The grain is partially amorphized, but some regions near the centre are crystalline with a structure consistent with orthopyroxene. The matrix in the section is dominated by phyllosilicates, magnetite, Fe sulfides and nanoglobules. Na-, C- and N-bearing material is also observed, including directly underneath grain 2b_2a.

**Extended Data Fig. 9:**
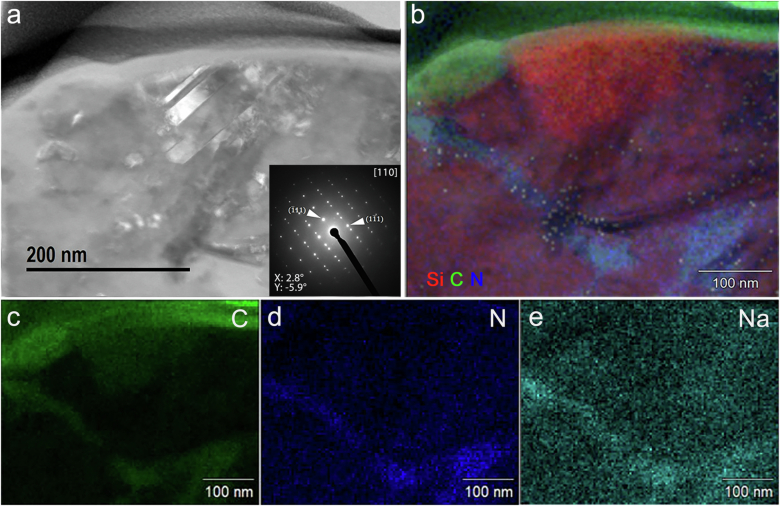
TEM analysis of a presolar SiC grain (2b_2b) in Clast 3 of particle OREX-803172-0. (**a**) DF-TEM image of the presolar grain under C and Pt caps which served to mark the grain. The inset is a [110] zone-axis SAED pattern demonstrating that the grain is a twinned 3C SiC. (**b**) Composite elemental map of the SiC. (**c–e**) C, N, and Na elemental maps showing a C-, N-, and Na-bearing vein.

The ^13^C-rich presolar grain 2b_2b from Clast 3 is ~225 × 130 nm and is a twinned SiC of the 3C polytype (Extended Data Fig. 9). The grain is surrounded by Mg-bearing silicate with adjacent Fe sulfides. Na-, C- and N-bearing material is observed in proximity to 2b_2b.

## Discussion

### Population of C-rich presolar grains in Bennu

Presolar SiC grains are the most extensively studied type of stardust grain, due, in part, to the ability to chemically isolate them from the bulk matrix and their occurrence as large (>1 μm) grains. Based on their C, N and Si isotopic compositions, these grains have been grouped into eight types^4,14^ (Fig. 2b). Mainstream grains are the most abundant type and derive from asymptotic giant branch (AGB) stars, as do type Z and Y grains. Type AB grains are characterized by greater ^13^C enrichments than mainstream grains and could come from J-type carbon stars, born-again AGB stars or supernovae. The AB grains with enrichments in ^15^N relative to solar composition are likely to have supernova origins^16,17^. Types X, C and D grains also have supernova origins. Type N grains come from novae or supernovae.

We classified the presolar SiC grains in Bennu based on published criteria^14^ and found all presolar SiC grain types, except for the rarest C, N and D grains. Most of the grains derive from AGB stars, in concurrence with previous observations of presolar SiC grains in chondritic meteorites and Ryugu^1,2,14^. However, the Bennu particles we analysed exhibit a higher proportion of the rare X and AB grains. All of the AB grains are ^15^N-rich relative to solar (Fig. 2b), making a supernova origin likely^16,17^. Combining our results with those of refs. ^6,13^, we report that the Bennu samples analysed thus far contain a significantly greater proportion of supernova-derived SiC grains (31%) relative to all other chondritic samples in which presolar SiC have been identified (5% (refs. ^14,16^)).

Studies of IDPs and AMMs, believed to derive from comets, indicate a possible enrichment in O-rich supernova grains^18–20^, and one of the three presolar SiC grains identified in cometary samples is a ^15^N-rich AB grain^21^. The elevated abundance of supernova dust in Bennu and cometary samples suggests that the formation regions of Bennu’s parent asteroid and comets were seeded by supernova dust that was not well mixed in the outer protoplanetary disk (beyond Jupiter’s current orbit at ~5 au) before accretion.

Presolar SiC and graphite are altered by heating in the nebula^22^, prolonged oxidation and thermal metamorphism on the parent body, leading to a reduction in their abundances^5,23^. Extended low-temperature heating oxidizes the outer portions of SiC grains to form SiO_2_. The abundances of presolar SiC in the matrix of the angular and hummocky particles and in the clasts are within error of each other (Extended Data Fig. 5 and Extended Data Table 1), with an overall abundance of ${45}_{-6}^{+7}$ ppm. This abundance is consistent with that in the main hydrated lithology of Ryugu^1,2^ and with most chondrites that have not been thermally metamorphosed; it is slightly higher than abundances in the CI Ivuna and the CM Murchison^2,5^. Bennu’s parent body may therefore have experienced lower temperatures or shorter heating duration than those of CI and CM chondrites, which is consistent with the low temperature (<50 °C) of aqueous alteration inferred from mineralogical observations of Bennu samples^12,24^.

The TEM analysis of presolar SiC grain 2b_2b (Extended Data Fig. 9) from Clast 3 does not show evidence of heating whereas grain 15c (Extended Data Fig. 6) has an oxidized rim. The high carbon abundance of Clast 1, in which grain 15c was found, and the lack of evidence for thermal metamorphism in surrounding matrix materials support minimal parent body heating. The oxidized rim on grain 15c was, therefore, likely to be the result of nebular heating, similar to a presolar SiC observed in an exogenous clast in Ryugu^1^.

### Preservation of O-rich presolar grains

The O-rich presolar grains in the Bennu samples mainly have ^17^O enrichments with slight ^18^O depletions that indicate origins in AGB stars or supernovae. Two grains, one ^17^O poor and one ^18^O rich, are likely to have supernova origins (Fig. 2a). Nova grains represent ~1% of presolar O-rich grains^4^, and one ^17^O-rich nova grain was identified in Clast 1. The majority (21 out of 25) of the O-rich presolar grains are silicates.

The survival of presolar silicates in astromaterials is sensitive to the degree of parent body aqueous alteration. Carbonaceous chondrites are classified based on their bulk petrographic characteristics and degree of alteration, with type 1 being the most extensively hydrated, type 3 the least altered, and types >3 are thermally metamorphosed^25^. CI chondrites are among the most chemically primitive samples, with chemical compositions closely resembling the solar photosphere^26^. However, they are all type 1—their parent asteroids have undergone extensive aqueous alteration, converting most of the original constituents to hydrous phases. Consequently, no surviving presolar silicates have been identified in CI chondrites^2^. Bennu and Ryugu are closely related to CI chondrites^13,27^, and with the spacecraft’s detection of substantial hydrated phases on Bennu, most presolar silicate grains were expected to have been destroyed during processing on the parent asteroid.

The abundances of O-rich presolar grains in the matrix of the angular and hummocky Bennu particles and most of the clasts (except for Clasts 1–3 (Fig. 1), discussed below) were within error of each other, for an overall abundance of 4 ± 2 ppm (Fig. 2c, Extended Data Fig. 4 and Extended Data Table 1). This indicates that these lithologies experienced a similar degree of aqueous alteration on the parent asteroid. The abundance of O-rich presolar grains is consistent with that reported for aggregate Bennu material (6 ppm)^13^ and for the major hydrated Ryugu lithology (3 ppm)^1,2^. These low abundances are indicative of considerable aqueous alteration, but they also show that both of the major Bennu lithologies identified to date, and Ryugu’s major hydrated lithology, are less altered than some type 1 chondrites. The ungrouped type 2 chondrite Tagish Lake, for example, has a similar abundance of O-rich presolar grains to these Bennu lithologies^28^.

### Characteristics of less-altered clasts

Most (72%) of the O-rich presolar grains are located within three S-rich clasts within the hummocky particle OREX-803172-0 (Clasts 1–3; Fig. 1). The higher abundances indicate greater survival of presolar silicates in these clasts compared with the angular particles, host hummocky matrix and other measured clasts. Therefore, we infer that Clasts 1–3 are less altered than the major lithologies of Bennu, Ryugu and CI chondrites. Their average abundance (${87}_{-20}^{+26}$ ppm) is consistent with those in an exogenous clast found in a Ryugu sample (104 ppm)^1^ and with primitive carbonaceous chondrites, such as Asuka 12236 (CM2.9; 58 ppm)^29^, Miller Range (MIL) 090019 (CO3; 77 ppm)^30^, Northwest Africa (NWA) 852 (CR2; 116 ppm)^31^, Meteorite Hills (MET) 00426 (CR2; 160 ppm)^32^ and Adelaide (C2-ungrouped; 70 ppm)^33^ (Fig. 2c and Extended Data Fig. 4). However, the Bennu clasts are dominated by phyllosilicates, whereas most of these type 2–3 chondritic samples retain amorphous material in the groundmass. For Clasts 1–3 to have similar abundances to these more primitive chondritic samples, they must have started off with higher initial abundances of O-rich presolar grains before aqueous alteration occurred.

**Fig. 5:**
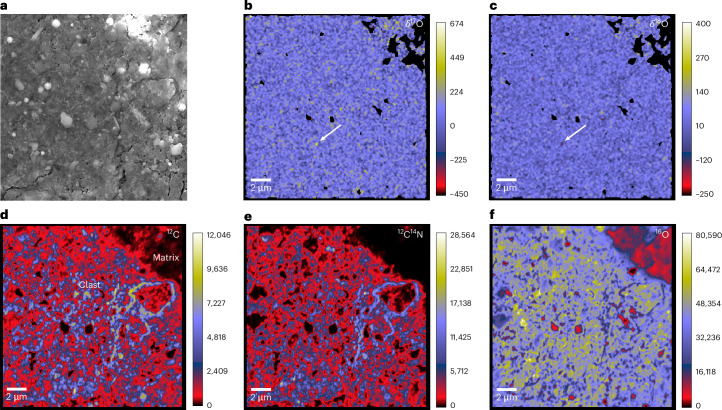
Isotope mapping of a region in hummocky particle OREX-803172-0 containing Clast 1. **a**, Secondary electron image of an area containing material from Clast 1 and the adjacent host matrix (labelled in d). **b**,**c**, Corresponding NanoSIMS O isotope ratio maps with arrows indicating a ^17^O-rich (**b**) and ^18^O-poor (**c**) presolar silicate grain in Clast 1. **d**–**f**, ^12^C (**d**), ^12^C^14^N (**e**) and ^16^O (**f**) ion images showing that the clast is richer in these isotopes than the surrounding matrix.

The NanoSIMS analysis of Clasts 1–3 shows that they are enriched in C and N compared with the other clasts and matrix (Fig. 5), with an average CN^−^/C^−^ ratio of 2.6 compared with 2.0 in the other S-rich clasts, 1.5 in the hummocky matrix and 0.9 in the angular matrix. High CN^−^/C^−^ ratios similar to those of Clasts 1–3 have been observed in purported cometary IDPs^34^. The carrier of this C and N is organic matter. Soluble organic matter in Bennu aggregate samples is also N rich^11^.

Although the exogenous clasts in Ryugu samples have similar chemical characteristics to the Bennu Clasts 1–3—being poor in Si and Mg and rich in S, Fe, C and N relative to the surrounding matrix—the CN^−^/C^−^ ratio (0.8) is three times lower and they are dominated by amorphous silicates, including glass with embedded metal and sulfide-like grains^1^. The presence of these glass with embedded metal and sulfide-like grains indicates a very low degree of aqueous alteration. Moreover, the exogenous clasts have extremely high abundances of presolar SiC; 235 ppm compared with 33 ppm in Bennu Clasts 1–3. This lower presolar SiC abundance in the Bennu clasts cannot be attributed to destruction by thermal metamorphism because the clast mineralogy shows that they were not thermally altered. Therefore, the exogenous clasts in Ryugu samples and Clasts 1–3 in the Bennu hummocky particle have disparate origins.

Clasts 1–3 show distinguishing features among themselves. All are decorated with anhydrous silicates (mainly olivine), but coarser grains (>5 μm) are not visible on the surface of Clast 3. Clast 3 is depleted in O and richer in C relative to Clasts 1 and 2 and shows a dehydrated texture with prominent cracks resembling those produced by thermal cycling^35^. Evidence for thermal cycling was also observed in boulders on Bennu^36^. However, this is at odds with the SiC abundance that indicates minimal thermal heating. The Na-, C- and N-bearing material in Clast 3 (Extended Data Figs. 8 and 9) is likely to have formed on Bennu’s parent body during late-stage evaporation of an alkaline brine^24^ that filled the cracks and fractures. Clast 2 is chemically heterogenous, with portions being more Fe and S rich due to the presence of pyrrhotite with a fibrous texture. The characteristics of Clasts 2 and 3 suggest that localized water–rock interactions occurred before their being emplaced into the surrounding matrix, which had been altered more extensively, as discussed in ref. ^10^. Alternatively, the clasts may have been protected from more extensive alteration; however, no conclusive evidence for this is observed.

The mineralogy of the presolar grains also supports incomplete hydration of the clasts. Stoichiometric Mg-rich silicate grains are predicted to condense in circumstellar environments from a gas of solar composition at 1,444 and 1,354 K assuming total pressures of 10^−3^ and 10^−4^ bar, respectively^26^. Non-stoichiometric, amorphous grains are likely to have formed through non-equilibrium processes^37^. Evidence for parent body alteration of presolar silicates in chondrites has been observed as amorphization and infiltration of Fe from the surrounding matrix^33,38–40^. Crystalline Mg-rich presolar forsterite grains have thus far only been identified in primitive carbonaceous chondrites and IDPs^19,38,41–43^. The presolar forsterite grain 16_2 from Bennu Clast 1 (Fig. 3) has a Mg-rich stoichiometric composition and maintains its primary crystalline structure, suggesting formation under equilibrium conditions around the parent star. The surrounding matrix is Fe rich, but infiltration into the presolar grain is not observed. The pyroxene grain 2b_2a is partially amorphized (Extended Data Fig. 8), and the Mg-rich chemical composition suggests that this amorphization occurred in space or the nebula rather than on the parent body, similar to a partly amorphized presolar enstatite grain found in the Queen Alexandra Range (QUE) 99177 meteorite^38^.

Presolar hibonite and spinel have previously been identified as individual grains in chondrites and an IDP^44–46^, but none share the same microstructure as the compound presolar spinel–hibonite grain 19_1 (Fig. 4). Equilibrium condensation calculations predict that hibonite is one of the first minerals to condense from a gas of solar composition, at 1,728 K and 1,659 K assuming total pressures of 10^−3 ^bar and 10^−4^ bar, respectively^26,47^. Spinel condenses at lower temperatures of 1,488 K and 1,397 K at 10^−3^ bar and 10^−4^ bar, respectively^26,47^. Therefore, the presolar spinel–hibonite assemblage is consistent with equilibrium condensation, where the hibonite formed first and spinel formed by back reaction of hibonite with the gas. The crystallographic orientation relationship between the spinel and hibonite in grain 19_1 indicates epitaxial nucleation and growth of spinel onto the hibonite upon cooling in the stellar outflow. Similar mineralogical relationships have been observed in hibonite–spinel refractory inclusions that condensed in the early solar nebula, found in the Allan Hills (ALHA) 77307 meteorite^48^. The condensation conditions in the nebula were therefore similar to those around the parent star of 19_1. The well-preserved crystal structure of presolar grains 16_2 and 19_1 indicate that they remained undisturbed following their formation.

Presolar grain 5_3b (Extended Data Fig. 7) is an amorphous silicate that exhibits small deviations from perfect stoichiometry and envelops a crystalline sulfide inclusion. Under equilibrium conditions, sulfides are predicted to condense at lower temperatures (704 K, 10^−4^ bar) than Mg–Fe silicates (1,316 K, 10^−4^ bar)^26^. Therefore, the amorphous non-stoichiometric nature of the silicate and the inclusion of a low-temperature phase is inconsistent with equilibrium condensation. The uniform chemical composition of the silicate and crystalline nature of the sulfide are also inconsistent with parent body alteration. Presolar grain 5_3b is likely to have formed under non-equilibrium conditions, which is consistent with previous reports of sulfides within silicate presolar grains^40,49^ and amorphous non-stoichiometric presolar silicates^37,50^.

### Implications for Bennu’s protolith

The results presented here for angular and hummocky Bennu particles suggest that both lithologies are less altered than type 1 chondrites, including CI, but more altered than type 3 chondrites, which is in agreement with observations of Bennu aggregate samples^11,13^. The presence of anhydrous minerals in the particles indicates mixing of material from the inner Solar System to the outer regions that had inherited organic matter and presolar grains. Bennu aggregate material also showed greater proportions of isotopically anomalous organics and anhydrous silicates than its closest counterparts, CI chondrites and Ryugu^13^. The Bennu angular and hummocky lithologies are distinct from other chondritic samples in their six-times higher abundance of supernova SiC grains, suggesting that the parent asteroid sampled material from a region of the outer protoplanetary disk enriched in supernova-derived SiC grains.

Clasts 1–3 are less altered, with very N-rich organic matter, presolar grains having wide-ranging mineralogy and likely had higher initial abundances of presolar silicates than type 3 chondrites. These attributes suggest that they are more akin to type 3 chondrites, and even some IDPs, making them the most primitive CI-like material characterized to date. Thus, although Bennu’s parent body was indeed heavily altered, pockets of less-altered material are preserved as intraparticle clasts that may represent the starting materials that accreted to form Bennu’s protolith. Previous work demonstrated that Bennu’s parent body shared an accretionary reservoir with those of CI chondrites and Ryugu^13^. Our findings indicate that the clasts accreted material from a region of the disk with a greater proportion of presolar silicate grains and N-rich organics than is typically observed in chondritic meteorites and Ryugu, which are compositional features more akin to some comets.

## Methods

### Sample preparation and chemical characterization

The samples studied were obtained from within the touch-and-go sample acquisition mechanism^51^. The parent sample was OREX-803017-0. Particles >500 μm were picked out and attached to C tape on Al cylinder mounts for characterization using the JEOL 7600F SEM at NASA JSC, equipped with Oxford Instruments Ultim Max EDX detectors. A 15-kV, ~1-nA beam was used to produce sample images, elemental maps and point spectra. Two polished particle mounts, OREX-803079-0 and OREX-803080-0, were subsequently produced by embedding individual particles in epoxy and polishing solvent free with diamond powders. Both particle mounts consisted of five particles. Conductive C coats were deposited onto the samples. SEM-EDX mapping was conducted on the polished particle surfaces and point spectra were obtained on individual minerals. Sample OREX-803079-0 was then polished further using the Hitachi ArBlade 5000 ion milling system.

### NanoSIMS isotopic analysis

Isotope mapping was performed using the NanoSIMS 50L at NASA JSC. Three particles from mounts OREX-803079-0 and OREX-803080-0 were targeted for these analyses: angular particles OREX-803165-0 and OREX-803170-0, and hummocky particle OREX-803172-0. The particle OREX-803172-0 fragmented during sample handling and both fragments, named ‘D1’ and ‘D2’ (Supplementary Tables 1 and 2), were analysed. An ~100-nm, 1-pA Cs^+^ primary ion beam was rastered over 20-μm fields of view. Each analysed region was presputtered using a high-current (~125 pA) primary beam to remove the conductive coating, implant Cs^+^ for improved ion yield and to ensure that the secondary ion yield reached a steady state. The ion images consisted of 256 × 256 pixels and multiple planes were acquired for each region.

OREX-803165-0 was first mapped for ^12^C, ^13^C, ^12^C^14^N, ^12^C^15^N, ^28^Si, ^29^Si and ^30^Si. The negative secondary ions of these isotopes were collected simultaneously in electron multipliers. A total area of 20,415 μm^2^, based on the summed ^12^C and ^28^Si images, was measured using this analytical set-up. USG24 graphite and terrestrial kerogen were used for instrument tuning and served as isotopic standards. San Carlos olivine was used for tuning Si isotopes, but the matrix in the sample was used for normalization. A mass resolving power of ~10,000 (CAMECA definition) allowed isobaric interferences to be resolved. The L’image software (https://sites.google.com/carnegiescience.edu/limagesoftware/) was used to process the isotope images and grains were considered presolar if their C, N or Si isotopic compositions deviated by >5*σ* from the standard ratio. Preliminary phase classifications were made based on the ^28^Si/^12^C ratios. Three anomalous SiC grains had N contents below the detection limit of the NanoSIMS.

In the second analytical set-up, ^12^C, ^13^C, ^12^C^14^N, ^12^C^15^N, ^16^O, ^17^O and ^18^O were measured simultaneously in all three Bennu particles. USG24 graphite, terrestrial kerogen and San Carlos olivine were used for instrument tuning and served as isotopic standards. Based on the summed ^12^C and ^16^O images, total areas of 24,301 μm^2^, 6,020 μm^2^ and 60,625 μm^2^ were analysed in OREX-803165-0, OREX-803170-0 and OREX-803172-0, respectively. Grains having C, N or O ratios >5*σ* from the standard ratios were considered presolar.

The presolar grains in OREX-803170-0 and OREX-803172-0 were remeasured at higher spatial resolution over 3–6-μm fields of view. C-rich presolar grains were measured for C and Si isotopes, and phase classifications were made based on the ^28^Si/^12^C ratios. The Si ion images were examined for grains having relatively low Si/C, and grains showing a clear signature for Si were classified as SiC and not graphite. Three additional presolar SiC grains were identified during these analyses and were therefore not measured for N isotopes. O-rich presolar grains were remeasured for O and Si isotopes, and phase classifications were made based on the ^28^Si/^16^O ratios. Grains with a clear signature for Si in the Si ion image were classified as silicates.

Presolar grain abundances (parts per million) were calculated as the summed area of a presolar phase (O-rich, SiC, graphite) over the total area analysed. Abundance errors of 1*σ* are reported. These errors are based on counting statistics and are calculated using the confidence limits tables in ref. ^52^. The abundances of O-rich and SiC presolar grains are compared with data from Ryugu^1,2^ and various carbonaceous chondrites^2,5,13,28–33,43,53–55^ in Fig. 2c and Extended Data Figs. 4 and 5 (see Source Data Fig. 2c and Source Data Extended Data Figs. 4 and 5 in the Supplementary Table for the source data).

### TEM analyses

To identify candidates for FIB extraction, presolar grains were imaged and their locations identified using the JEOL 7900F SEM at JSC. Six grains were targeted for chemical and structural analysis by TEM. Electron transparent cross-sections (OREX-803172-100, OREX-803080-102, OREX-803080-103, OREX-803080-104, OREX-803080-105) were produced using established FIB-SEM methods^38^. Importantly, C and Pt markers were placed on top of the presolar grains to ensure that they could be identified during thinning of the sections and during TEM analysis. FIB sections were analysed at 200 keV using the JEOL 2500SE STEM at JSC. The JEOL 2500SE is equipped with bright-field, dark-field and secondary electron STEM imaging detectors, a JEOL 60-mm^2^ silicon drift detector for EDX analyses and mapping and a Gatan OneView 4 k × 4 k pixel CMOS camera for high-resolution TEM imaging and electron diffraction. Grain compositions were obtained by summing regions of interest from EDX spectrum images and quantifying the spectra using the Cliff–Lorimer procedure with experimentally determined *k*-factors.

## Supplementary information

Supplementary Information Supplementary Tables 1–3 and source data for Fig. 2c and Extended Data Figs. 4 and 5.

## Figures and Tables

**Extended Data Table 1:**
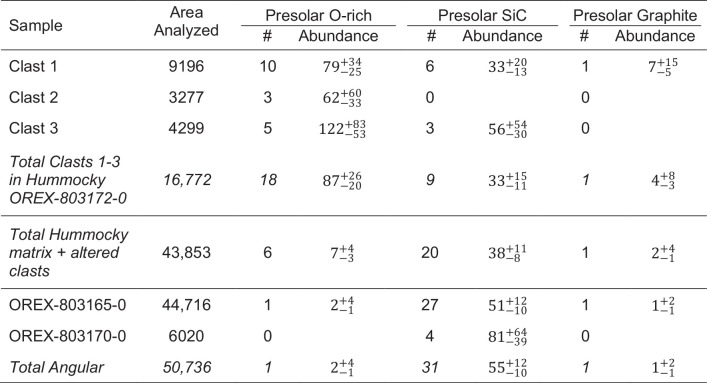
Presolar grain abundances in Bennu hummocky and angular particles

Area analyzed is given in square microns. The number (#) symbol denotes the number of presolar grains identified. Abundances are given as parts per million (ppm) and errors are 1σ. For the hummocky particle, we report the abundances for Clasts 1–3 separately from those of the matrix and other clasts.

## Data Availability

Instrument data that support the findings of this study are available via astromat.org; see Supplementary Data Table 3 for DOIs.
